# A11 WAIT TIMES FOR COLONOSCOPY ACCORDING TO SUBSEQUENT PATHOLOGY TO IMPROVE A COLONOSCOPY REFERRAL SHEET

**DOI:** 10.1093/jcag/gwaf042.011

**Published:** 2026-02-13

**Authors:** S Aprikian, C Hansen-Barkun, N Milky, K Shanahan, J Beauchesne-Blanchet, M Martel, C Menard, D Von Renteln, A N Barkun

**Affiliations:** McGill University, Montreal, QC, Canada; McGill University, Montreal, QC, Canada; McGill University, Montreal, QC, Canada; Research Institute of the McGill University Health Centre, Montreal, QC, Canada; Research Institute of the McGill University Health Centre, Montreal, QC, Canada; Research Institute of the McGill University Health Centre, Montreal, QC, Canada; Universite de Sherbrooke, Sherbrooke, QC, Canada; Universite de Montreal, Montreal, QC, Canada; McGill University, Montreal, QC, Canada

## Abstract

**Background:**

The use of Quebec’s provincial colonoscopy referral form (FRCQ), which groups clinical indications according to prioritized wait-time categories, aims to optimize access to colonoscopy for patients presenting with clinically significant lesions (CSL), including colorectal cancer (CRC). However, although the prioritization of requests is based on the clinical indication, the actual waiting times observed according to pathological diagnoses remain poorly documented. Such an evaluation would allow a more direct validation of the relevance of the FRCQ as a provincial triage tool.

**Aims:**

To assess wait times from referral to colonoscopy according to findings on pathology, with a focus on CRC and other CSL including and excluding polyps, and considering active colitis.

**Methods:**

This retrospective cohort study included consecutive adult patients referred through the Quebec province-wide colonoscopy referral form (PCRF) from two tertiary hospitals. The primary outcome was days from initial referral request to the time of the colonoscopy (wait time) according to corresponding pathology lesions at the index colonoscopy categorized as CRC, CSL (that include cancer, advanced adenomas, serrated lesions, colitis, strictures, other clinically relevant findings), CSL without polyps, and sole active colitis.

**Results:**

Overall, 14,656 patients (mean age 59.2 ± 14.0 years, 50.9% female) were included over a 47-month period; the overall wait times was 126.5 ± 199.5 days. Patients with no CSL (N = 8901) waited less than those with CSL (120.0 ± 185.1 days vs 136.0 ± 219.6, p < 0.01). Patients eventually found to have a colorectal cancer at colonoscopy (N = 127) were endoscoped within the shortest wait times compared to all other individuals referred for colonoscopy (59.0 ± 115.6 vs 127.1 ± 200.0 days, p < 0.01). Wait times were significantly longer for patients found to have polyps (N = 5841) compared to patients without polyps (139.5 ± 226,7 vs 117.9 ± 178.7, p < 0.01). Among CSL subgroups, patients with active colitis (N = 417) had similar wait times compared to those without (139.4 ± 170.9 vs 126.1 ± 200.2 days, p = 0.18).

**Conclusions:**

Overall wait times for colonoscopy in this large sample of patients in the province of Quebec are in keeping with suggested standards. These are appropriately shorter for patients found to have CRC, supporting current referral prioritization strategies. However, among non-cancer CSL findings, delays are not discriminant once patients with polyps, who for the most part are asymptomatic, are excluded. These findings highlight the need for refined predictors to further optimize the colonoscopy provincial referral triage sheet, especially when considering non cancer subgroups, and in particular patients eventually found to have an acute colitis.

**Funding Agencies:**

CPAC

